# Assessing the Long-Term Impact of the COVID-19 Pandemic on Hospital Outcomes in Patients with Decompensated Liver Cirrhosis

**DOI:** 10.3390/medicina62020404

**Published:** 2026-02-19

**Authors:** Melania Veronica Ardelean, Ovidiu Florin Ardelean, Dana Roxana Buzas, Paul Ciubotaru, Vlad Ivan, Alin Viorel Istodor, Daniel Florin Lighezan, Norina Simona Basa

**Affiliations:** 1 Department V, Internal Medicine I Discipline of Medical Semiology I, “Victor Babes” University of Medicine and Pharmacy, Eftimie Murgu Sq. No 2, 300041 Timisoara, Romania; ardelean.melania@umft.ro (M.V.A.); paul.ciubotaru@umft.ro (P.C.); ivan.vlad@umft.ro (V.I.); dlighezan@umft.ro (D.F.L.); basa.simona@umft.ro (N.S.B.); 2 Center of Advanced Research in Cardiology and Hemostasology, “Victor Babes” University of Medicine and Pharmacy, Eftimie Murgu Sq. No 2, 300041 Timisoara, Romania; 3 Department IX, II Discipline of Surgical Semiology, “Victor Babes” University of Medicine and Pharmacy, Eftimie Murgu Sq. No 2, 300041 Timisoara, Romania; 4 I Department of Surgery, I Discipline of Surgical Semiology and Thoracic Surgery, “Victor Babes” University of Medicine and Pharmacy, Eftimie Murgu Sq. No 2, 300041 Timisoara, Romania; istodor.alin@umft.ro; 5 Center Hepatobiliary Pancreatic Surgery, “Victor Babes” University of Medicine and Pharmacy, Eftimie Murgu Sq. No 2, 300041 Timisoara, Romania

**Keywords:** liver cirrhosis, COVID-19 pandemic, healthcare disruption, mortality predictors, MELD and Child–Pugh scores, endoscopy access, post-pandemic recovery

## Abstract

*Background and Objectives*: The COVID-19 pandemic profoundly disrupted global healthcare systems, limiting access to diagnostic and therapeutic services for chronic diseases. Patients with decompensated liver cirrhosis were particularly vulnerable due to their fragile clinical status and dependence on continuous medical care. This study aimed to evaluate the temporal evolution of clinical, biological, and prognostic parameters in patients admitted emergently with decompensated liver cirrhosis across three distinct phases: pre-pandemic, pandemic, and post-pandemic. *Materials and Methods*: A retrospective, single-center study was conducted at the Department of Gastroenterology, Municipal Clinical Emergency Hospital, Timișoara, Romania, including 355 patients hospitalized between February 2018 and February 2024. Clinical, biochemical, and outcome data were collected and analyzed using univariate and multivariate logistic regression models to identify independent predictors of in-hospital mortality for each study period. *Results*: Significant temporal variations were observed in disease severity, management, and outcomes. The mean MELD score increased from 18.7 to 21.0 during the pandemic (*p* = 0.043), while endoscopic evaluations declined markedly (59.4% pre-pandemic vs. 42.7% pandemic, *p* = 0.037). Mortality rose from 21.7% to 30.2% during the pandemic (*p* = 0.044) and remained elevated post-pandemic (26.4%). Multivariate regression identified Child–Pugh, MELD, and Baveno scores as consistent mortality predictors, though their relative weight varied by period. During the pandemic, acute complications—particularly jaundice (OR = 294) and upper gastrointestinal bleeding (OR = 355)—became dominant determinants of death. *Conclusions*: The pandemic transformed cirrhosis from a chronic, manageable disease into an acutely unstable condition, primarily due to delayed presentation and restricted procedural access. Although post-pandemic recovery was evident, residual increases in mortality and severity indicate lasting effects of healthcare disruption, underscoring the need to strengthen system resilience and continuity of care for patients with chronic liver disease.

## 1. Introduction

Liver cirrhosis represents one of the leading causes of liver-related mortality worldwide, accounting for approximately two million deaths annually—about half resulting from direct complications of the disease, while the remainder are attributable to secondary hepatocellular carcinoma [1,2]. In Europe, the prevalence of cirrhosis is estimated at 0.3%; however, in Eastern Europe, both incidence and mortality rates are markedly higher due to the increased prevalence of viral hepatitis B and C, as well as excessive alcohol consumption [3]. In Romania, recent data indicate a mortality rate of 33.6 per 100,000 inhabitants, placing the country among the leading European Union members in deaths attributed to chronic liver diseases [4].

Etiologically, the leading causes of liver cirrhosis include chronic viral infections (HBV and HCV), prolonged alcohol consumption, and non-alcoholic steatohepatitis. In Romania, alcoholic cirrhosis remains predominant, being identified in over 70% of hospitalized cirrhotic patients [4,5]. Nevertheless, therapeutic advances introduced by direct-acting antivirals (DAAs) for hepatitis C, together with improvements in diagnostic strategies and overall cirrhosis management, have substantially reduced the incidence of severe complications such as decompensated cirrhosis. In parallel, recent studies have highlighted the broader clinical impact of optimized cirrhosis care, including improvements in patient-related outcomes and quality of life, reinforcing the importance of timely diagnosis and comprehensive disease management [5,6]. A multicentric European study that included Romania demonstrated that DAA therapy prevented between 1000 and 1200 cases of decompensated cirrhosis and hepatocellular carcinoma per 1000 treated patients, leading to healthcare cost reductions of up to €275 million [4].

The COVID-19 pandemic generated an unprecedented global crisis that profoundly disrupted healthcare systems worldwide [7,8,9]. Within a remarkably short period, hospitals were compelled to reallocate human and material resources toward the care of patients infected with SARS-CoV-2, thereby severely limiting access to medical services for individuals with chronic conditions [8,9,10]. Multiple international studies have demonstrated that the pandemic led to a decline in non-urgent hospital admissions, the suspension of elective procedures, and the postponement of scheduled treatments—particularly in fields such as gastroenterology, oncology, and liver transplantation [11,12,13,14].

In Romania, the impact of the COVID-19 pandemic on the management of liver cirrhosis closely mirrored the global disruptions in hepatology care. During the pandemic period, patients with liver cirrhosis experienced significantly restricted access to routine monitoring, including laboratory investigations and endoscopic surveillance, as well as limited availability of emergency diagnostic and therapeutic procedures. In parallel, liver transplantation activity was temporarily reduced between 2020 and 2022, further constraining therapeutic options for patients with advanced liver disease [9,15]. These systemic limitations translated into delayed hospital presentation and more advanced clinical decompensation at admission. At a university medical center in Timișoara, hospitalizations for cirrhosis-related complications declined by approximately 30% during the pandemic, reflecting both reduced healthcare accessibility and patients’ reluctance to seek medical care. Importantly, this reduction in admissions was accompanied by a marked deterioration in disease severity at presentation, with significant increases in both MELD and Child–Pugh scores, indicating delayed access to care and a higher burden of advanced hepatic dysfunction at the time of hospitalization [9].

This situation was not an isolated occurrence. In Italy, Spain, and the United Kingdom, hepatology centers reported up to a 50% reduction in the number of endoscopic procedures performed in patients with cirrhosis, as semi-urgent interventions were postponed to minimize the risk of nosocomial infection [16,17,18]. Moreover, the intensive allocation of healthcare resources to the management of COVID-19 cases—including intensive care units, mechanical ventilation equipment, and specialized medical staff—substantially limited the capacity to treat patients with severe hepatologic conditions [6,9,11]. At the same time, patients’ fear of contracting the virus in hospital settings led to significant delays in seeking medical care, resulting in late presentations and severe, difficult-to-manage acute complications [19,20].

Despite multiple reports describing the immediate impact of the COVID-19 pandemic on hepatology services and cirrhosis outcomes, data evaluating the longitudinal evolution of disease severity and mortality predictors across pre-pandemic, pandemic, and post-pandemic phases remain limited, particularly in Eastern European settings.

Given the profound impact of the COVID-19 pandemic on healthcare systems and previous evidence demonstrating major changes in the clinical profile and prognosis of patients with liver cirrhosis, the aim of this study was to comparatively evaluate the evolution of clinical, biological, and prognostic parameters in patients admitted emergently with decompensated liver cirrhosis across three distinct periods: pre-pandemic, pandemic, and post-pandemic. Specifically, we sought to assess variations in disease severity, the prevalence of major complications, and independent predictors of in-hospital mortality using multivariate logistic regression models. By integrating these analyses, the study aims to clarify how pandemic-related disruptions in hepatology care influenced patient outcomes and to provide insight into the longer-term consequences for cirrhosis management in the post-pandemic period.

## 2. Materials and Methods

### 2.1. Study Design and Population

This retrospective, observational, single-center cohort study was conducted in the Department of Gastroenterology of the Municipal Clinical Emergency Hospital, Timișoara, Romania. It included all adult patients admitted as emergencies with a principal diagnosis of decompensated liver cirrhosis. Clinical, biochemical, and demographic data were collected retrospectively from electronic medical records and analyzed systematically.

The study period extended from 26 February 2018 to 25 February 2024 and was divided into three distinct intervals to capture the pandemic’s systemic influence on patient management:

- Pre-pandemic period: 26 February 2018–25 February 2020;
- Pandemic period: 26 February 2020–25 February 2022;
- Post-pandemic period: 26 February 2022–25 February 2024.

The choice of these intervals was deliberate. The first COVID-19 case in Romania was confirmed on 26 February 2020, marking the beginning of pandemic restrictions, while all official restrictions were lifted on 8 March 2022, thus providing comparable time frames for each study phase.

### 2.2. Inclusion and Exclusion Criteria

Patients were included if they met the following criteria:

- Confirmed diagnosis of liver cirrhosis (regardless of etiology);
- Emergency hospital admission during the defined study periods;
- No prior or active COVID-19 infection at admission;
- Absence of typical COVID-19 symptoms within seven days before presentation.
- Only patients with complete datasets were included in the final analysis.

Exclusion criteria:

- Positive COVID-19 RT-PCR test or typical clinical presentation suggestive of SARS-CoV-2 infection;
- Presence of hepatocellular carcinoma (HCC), confirmed clinically, radiologically, or histologically.
- Incomplete data sets.

Given the well-established adverse prognostic impact of SARS-CoV-2 infection in cirrhotic patients, individuals with a positive COVID-19 test or typical clinical presentation were excluded, as they were admitted to designated isolation units and managed according to separate institutional protocols. After applying these criteria, a final cohort of 355 patients was retained for analysis.

### 2.3. Clinical Variables and Parameters

The study evaluated multiple domains:

Demographic characteristics: age, sex, and residential environment (urban/rural).

Presenting symptoms: abdominal pain, upper gastrointestinal bleeding (UGIB), defined as hematemesis and/or melena documented clinically and/or endoscopically; lower gastrointestinal bleeding (LGIB), defined as rectorrhagia or hematochezia, jaundice (self-reported by patients as “yellowing of the skin or eyes), lower limb edema (LLE), or other complaints (frequencies <5).

Etiology of cirrhosis: classified as alcoholic, viral, mixed, or other causes.

Severity indices:

- Child–Pugh score;
- Model for End-Stage Liver Disease (MELD) score—calculated as MELD = 9.57 × ln(creatinine) + 3.78 × ln(bilirubin) + 11.2 × ln(INR) + 6.43.
- Baveno staging system.

Markers of decompensation: presence of ascites, splenomegaly, esophageal varices, hepatic encephalopathy, thrombocytopenia, and the need for paracentesis.

Procedural indicators: necessity of therapeutic endoscopy (e.g., variceal band ligation, hemostasis interventions).

Supportive management: transfusion requirements, admission to the Intensive Care Unit (ICU), and duration of hospitalization.

Economic parameters: total hospitalization cost per patient.

Outcome variable: in-hospital mortality.

### 2.4. Ethical Considerations

This retrospective observational study was conducted using exclusively anonymized data extracted from existing electronic medical records of patients hospitalized between February 2018 and February 2024. The study was conceived after completion of the data accrual period. During the preparatory phase, the research question, study design, variables of interest, and statistical analysis plan were defined without access to identifiable patient information.

The study protocol was approved by the Ethics Committee of the Municipal Clinical Emergency Hospital Timișoara (Approval No. E-178/19 January 2026) and conducted in full accordance with the Declaration of Helsinki and national regulations on biomedical research involving human subjects.

### 2.5. Statistical Analysis

All statistical analyses were performed using IBM SPSS Statistics version 25.0 (IBM Corp., Armonk, NY, USA). A *p* value < 0.05 was considered statistically significant. Continuous variables were expressed as the mean ± standard deviation (SD), while categorical variables were summarized as absolute values and percentages.

The distribution of continuous variables was assessed using the Shapiro–Wilk test. For normally distributed data, comparisons between two groups employed Student’s *t*-test, while comparisons across more than two groups used one-way ANOVA, accompanied by Levene’s test for homogeneity of variances and Bonferroni correction for multiple comparisons. Non-normally distributed data were analyzed using the Mann–Whitney U test or Kruskal–Wallis test, as appropriate.

Categorical variables were compared using the Chi-square test or Fisher’s exact test when expected cell frequencies were <5. Continuous variables were not arbitrarily categorized unless clinically justified. Missing data were handled through listwise deletion, without imputation.

### 2.6. Multivariate Logistic Regression Modeling

To identify independent predictors of in-hospital mortality, a multivariate logistic regression analysis was performed. Variables were selected for multivariate analysis based on clinical relevance and statistical significance in univariate analysis (*p* < 0.05). Independent variables considered included: Child–Pugh score and MELD score (were treated as continuous variables), Baveno stage, jaundice, upper and lower gastrointestinal bleeding, ascites, abdominal pain, splenomegaly, and paracentesis requirement. Prior to model construction, potential multicollinearity between independent variables was assessed using correlation analysis, and no significant collinearity was identified. Model calibration was evaluated using the Hosmer–Lemeshow goodness-of-fit test. Odds ratios (ORs) with 95% confidence intervals (CIs) were reported to quantify the strength of associations. Separate regression models were generated for each period (pre-pandemic, pandemic, and post-pandemic) and for the combined cohort to assess temporal shifts in mortality predictors.

All results are reported with corresponding *p*-values and 95% CIs. Graphical visualizations were generated to illustrate variations in severity indices and mortality across study periods.

## 3. Results

Within the Department of Gastroenterology at the Municipal Hospital of Timișoara, a total of 355 patients were admitted on an emergency basis with a primary diagnosis of liver cirrhosis between 26 February 2020, and 25 February 2024. Statistically significant variations were observed in the number of admissions across the three study periods (*p* = 0.023). Specifically, 138 patients (38.9%) were hospitalized during the pre-pandemic interval, 96 (27.0%) during the pandemic, and 121 (34.1%) in the post-pandemic phase.

No significant differences were identified among the three periods regarding demographic characteristics, including sex, age, and area of residence. The detailed distribution of these variables is summarized in Table 1.

More than half of the patients presented with abdominal pain upon admission, totaling 191 individuals (53.8%). Among these, 73 patients (52.9%) reported this symptom during the pre-pandemic period, 55 (57.3%) during the pandemic, and 63 (52.1%) in the post-pandemic interval. No statistically significant differences were observed between the three periods (*p* = 0.718).

Similarly, no significant variations were found in the proportions of patients admitted for other clinical reasons such as upper or lower gastrointestinal bleeding, jaundice, or lower limb edema. The corresponding data are summarized in Table 2.

Hepatic encephalopathy, as a complication of cirrhosis, remained relatively stable throughout the three study periods, being observed in 84 patients (60.9%) during the pre-pandemic phase, 61 patients (63.5%) during the pandemic, and 74 patients (61.2%) in the post-pandemic interval (*p* = 0.908).

Significant differences were, however, noted in the proportion of patients presenting with thrombocytopenia (*p* = 0.020). During the pandemic, 47 patients (49.0%) exhibited low platelet counts, compared with 82 patients (59.4%) in the pre-pandemic period and 82 patients (67.8%) in the post-pandemic phase.

The etiology of cirrhosis did not show statistically significant variation across the three time intervals. Detailed data are provided in Table 3.

The presence of splenomegaly did not vary significantly across the three study periods (*p* = 0.767). During the pre-pandemic phase, 97 patients (40.1%) exhibited splenic enlargement, decreasing to 65 patients (26.9%) during the pandemic and subsequently rising slightly to 80 patients (33.1%) in the post-pandemic interval.

A similar trend was observed among patients presenting with esophageal varices; however, in this case, the differences were statistically significant (*p* = 0.016). The highest proportion was recorded before the pandemic, with 71 patients (51.4%), followed by the post-pandemic period with 59 patients (48.8%), while the lowest prevalence occurred during the pandemic, with only 32 patients (33.3%).

The occurrence of ascites reached its peak during the pandemic, affecting 75 patients (78.1%), compared with 101 patients (73.2%) in the pre-pandemic phase and 76 patients (62.8%) afterward. Statistical analysis confirmed significant differences among the three periods (*p* = 0.036). Nevertheless, the proportion of patients requiring paracentesis remained stable (30 (21.7%) vs. 23 (24.0%) vs. 27 (22.3%), *p* = 0.921).

Regarding endoscopic assessment, significant differences were identified in the proportion of patients undergoing gastroscopy during hospitalization (*p* = 0.037). The lowest rate was observed during the pandemic (42.7%), compared with 59.4% in the pre-pandemic interval and 55.4% in the post-pandemic interval. However, the need for interventional endoscopic procedures did not vary significantly across periods (*p* = 0.302).

Cirrhosis severity, assessed using the Child–Pugh classification, showed significant differences across the three periods, with a higher proportion of patients categorized as severe during the pandemic. Detailed data are summarized in Table 4.

An analysis of the Child–Pugh score was also performed, revealing statistically significant differences in the mean values across the three study periods (9.3 ± 2.46 vs. 9.92 ± 2.34 vs. 9.04 ± 2.26, *p* = 0.047).

In contrast, the Baveno score used to assess cirrhosis severity did not show significant variation between the evaluated time intervals. The detailed results are summarized in Table 5.

Significant differences were observed in the variation in the mean MELD score across the three study periods (*p* = 0.043). The average MELD value increased from 18.72 ± 8.88 in the pre-pandemic phase to 21.03 ± 10.19 during the pandemic and remained at a similarly elevated level in the post-pandemic period (21.04 ± 8.78).

### 3.1. Hospitalization

The duration of hospitalization represented one of the key parameters analyzed. Across the three study periods, the length of stay varied significantly (*p* = 0.007), decreasing from 6.88 ± 4.01 days in the pre-pandemic phase to 5.66 ± 4.51 days during the pandemic, and further to 5.47 ± 3.09 days in the post-pandemic period.

The need for blood transfusions during hospitalization also fluctuated. The proportion of patients receiving transfusions was 37.7% (52 patients) before the pandemic, dropped to 34.4% (33 patients) during the pandemic, and rose markedly to 47.1% (57 patients) afterward. A statistically significant difference was observed between the pandemic and post-pandemic periods (*p* = 0.048).

Similarly, ICU transfer requirements changed over time. The lowest rate was recorded in the pre-pandemic phase (6 patients, 4.3%), increasing to 10 patients (10.4%) during the pandemic and reaching 15 patients (12.5%) post-pandemic, a difference that proved statistically significant (*p* = 0.049).

Average hospitalization costs were estimated at €791 ± 461 in the pre-pandemic period, rising to €893 ± 437 during the pandemic, and remaining elevated in the post-pandemic years (€840 ± 391). Overall, the pandemic was associated with an average cost increase of 12.9% compared with the pre-pandemic phase and 6% higher than in the post-pandemic period.

Among all admitted patients, 87 individuals (24.5%) died during hospitalization. Mortality was lowest before the pandemic (26 patients, 18.8%), peaked during the pandemic (29 patients, 30.2%), and decreased slightly post-pandemic (32 patients, 26.4%). Statistical analysis confirmed a significant increase in in-hospital mortality during the pandemic (*p* = 0.044).

### 3.2. Mortality During the 3 Periods

To identify the independent predictors of in-hospital mortality among patients with decompensated liver cirrhosis, a multivariate logistic regression model was applied.

Variables with clinical relevance or statistical significance in the univariate analysis were included in the model, including the Child–Pugh and MELD scores, Baveno stage, and major complications such as jaundice (the reason patients presented to the hospital), esophageal varices, upper gastrointestinal bleeding, lower gastrointestinal bleeding ascites, abdominal pain, need for paracentesis and splenomegaly. The analysis aimed to determine which parameters independently contributed to mortality risk after adjusting for confounding factors.

The multivariate logistic regression analysis identified several independent predictors significantly associated with in-hospital mortality in all the patients (from all 3 periods) with decompensated liver cirrhosis:

- Child–Pugh score: B = 0.409, *p* = 0.001, OR = 1.505 (95% CI: 1.172–1.933).
  Patients with higher Child–Pugh scores had a 1.5-fold increase in mortality risk for each point increase.
- Baveno stage: B = 2.519, *p* < 0.001, OR = 12.421 (95% CI: 5.620–27.452).
  Patients in higher Baveno stages had a 12-fold higher risk of death, consistent with more severe portal hypertension and decompensation.
- MELD score: B = 0.107, *p* < 0.001, OR = 1.113 (95% CI: 1.050–1.180).
  Each additional MELD point was associated with an 11% increase in the odds of death.
- Splenomegaly: B = 1.295, *p* = 0.012, OR = 3.652 (95% CI: 1.330–10.029).
  The presence of splenomegaly was associated with a 3.6-fold higher risk of mortality.
- Abdominal pain: B = 1.462, *p* = 0.002, OR = 4.316 (95% CI: 1.690–11.023).
  Patients presenting with abdominal pain had a fourfold increased risk of in-hospital death.
- Paracentesis performed: B = 1.688, *p* = 0.010, OR = 5.410 (95% CI: 1.503–19.474).
  The requirement for paracentesis was associated with a fivefold higher mortality risk, indicating severe decompensation.
- Jaundice (the reason for hospital presentation): B = 3.642, *p* < 0.001, OR = 38.161 (95% CI: 6.487–224.492).

The complete results are presented in the Supplementary Material, Table S1, which summarizes the independent predictive factors for in-hospital mortality across all three study periods.

Given the significant temporal differences observed in both clinical and systemic variables, a separate multivariate regression analysis was performed for each study period. This approach was necessary to determine whether the influence of specific risk factors on mortality varied across distinct healthcare contexts—pre-pandemic, pandemic, and post-pandemic. By isolating each timeframe, the analysis allowed for a more accurate evaluation of how shifts in hospital capacity, access to diagnostic procedures, and disease severity patterns may have altered the prognostic weight of individual parameters, providing a clearer understanding of the dynamic relationship between patient characteristics and outcomes over time.

When analyzing the individual cohorts, the following findings were observed within the pre-pandemic group:

- Child–Pugh score: B = 0.565, *p* = 0.031, OR = 1.759 (95% CI: 1.053–2.939).
  Patients with higher Child–Pugh scores had a 1.7-fold increase in mortality risk per point increase.
- Baveno stage: B = 3.074, *p* = 0.002, OR = 21.624 (95% CI: 3.170–147.489).
  Patients in higher Baveno stages showed a 21-fold increase in the risk of death, indicating more advanced disease stages.

The complete results are presented in Supplementary Material, Table S2. Independent predictive factors for in-hospital mortality for pre-pandemic period.

When it came to the pandemic period, the statistically significant findings were as follows:

- Baveno stage: B = 3.411, *p* = 0.001, OR = 30.300 (95% CI: 4.066–225.766).
  Patients in higher Baveno stages had a 30-fold increased risk of death.
- MELD score: B = 0.159, *p* = 0.042, OR = 1.173 (95% CI: 1.006–1.368).
  Each additional MELD point was associated with a 17% increase in mortality odds.
- Upper gastrointestinal bleeding: B = 5.873, *p* = 0.003, OR = 355.287 (95% CI: 6.946–18,171.812).
  The occurrence of upper GI bleeding was associated with a markedly increased risk of death.
- Jaundice (the reason for hospital presentation): B = 5.684, *p* = 0.006, OR = 293.981 (95% CI: 4.933–17,520.171).

The presence of jaundice increased mortality risk by nearly 300 times.

The complete results are presented in Supplementary Material Table S3. Independent predictive factors for in-hospital mortality for pandemic period.

Regarding the post-pandemic period, the results were as follows:

- Child–Pugh score: B = 0.757, *p* = 0.008, OR = 2.131 (95% CI: 1.214–3.742).
  Patients with higher Child–Pugh scores had a twofold increase in mortality risk per point increase.
- Baveno stage: B = 3.463, *p* < 0.001, OR = 31.928 (95% CI: 5.002–203.796).
  Higher Baveno stages were associated with a 32-fold higher risk of death.
- MELD score: B = 0.147, *p* = 0.018, OR = 1.158 (95% CI: 1.025–1.309).
  Each additional MELD point increased mortality odds by approximately 16%.
- Lower gastrointestinal bleeding: B = 5.875, *p* = 0.022, OR = 355.896 (95% CI: 2.345–54,010.904).

The complete results are presented in Supplementary Material Table S4. Independent predictive factors for in-hospital mortality for post-pandemic period

## 4. Discussion

The COVID-19 pandemic profoundly disrupted global healthcare systems, forcing hospitals to redirect medical personnel, intensive care capacity, and diagnostic resources toward managing infected patients [13]. This reallocation led to a significant decline in hospital admissions for non-COVID pathologies, including cardiovascular, oncologic, and gastrointestinal conditions, as documented in several European reports [15,21,22,23]. Elective procedures were postponed, outpatient follow-ups were suspended, and preventive care was widely interrupted [17,24,25]. For patients with chronic liver disease—especially those with decompensated cirrhosis—these systemic pressures translated into delayed hospital presentations, advanced disease stages at admission, and increased mortality [9,26]. Endoscopic procedures and transplant programs were curtailed, while many individuals avoided hospitals due to fear of contacting the SARS-CoV-2 virus [17,27]. Consequently, cirrhosis-related complications such as variceal bleeding, ascites, and hepatic encephalopathy were often managed late, underscoring how the pandemic amplified existing vulnerabilities in hepatology care and exposed critical weaknesses in chronic disease management infrastructure [9,15].

### 4.1. Clinical Profile and Healthcare Impact Across the Three Periods

In the present cohort of 355 patients hospitalized emergently for decompensated cirrhosis, clear temporal variations were identified across the pre-pandemic, pandemic and post-pandemic intervals. Hospital admissions declined sharply during the pandemic, followed by only a partial recovery in subsequent years. This trend reflects international data showing that lockdown measures and reduced healthcare accessibility contributed to both underdiagnosis and delayed presentation among patients with chronic liver disease [28,29]. Such disruptions not only limited timely management but also altered the clinical spectrum of decompensated cirrhosis, as patients frequently presented with more advanced stages of disease, consistent with global observations during the COVID-19 crisis [13,15,29].

Importantly, although demographic characteristics such as age, gender, and environment remained comparable across the analyzed intervals, the clinical condition at admission deteriorated significantly during the pandemic, as reflected by higher MELD and Child–Pugh scores and an increased proportion of patients presenting with advanced Baveno stages. The marked decline in endoscopic assessments (42.7% during the pandemic vs. 59.4% pre-pandemic, *p* = 0.037) illustrates the systemic burden and the widespread postponement of elective and semi-urgent interventions [12,17]. These findings parallel those reported in Italian, Spanish, and UK hepatology centers, where endoscopic procedures in cirrhotic patients dropped by up to 50%, resulting in greater hospital mortality and delayed management of acute complications [16,17,18].

Furthermore, the progressive reduction in hospitalization duration (from 6.88 to 5.66 days, *p* = 0.007) observed during the pandemic was largely driven by physicians’ intention to minimize patients’ exposure to hospital-acquired SARS-CoV-2 infection. Infection control protocols and restrictive ward policies were implemented to reduce nosocomial transmission risks, indirectly leading to earlier discharges and shorter hospital stays among patients with decompensated cirrhosis [30,31]. Although post-pandemic data indicate a partial recovery in admission rates and procedural activity, essential clinical outcomes—such as mortality and MELD scores—remained elevated compared with the pre-pandemic period. This suggests that the consequences of limited hepatology access and postponed interventions persisted well beyond the crisis, reflecting long-term effects on patient outcomes and overall disease progression [3,32].

### 4.2. Disease Severity, Complications, and the Evolving Clinical Landscape

The descriptive and comparative analyses clearly indicate a shift in the clinical spectrum of decompensated cirrhosis across the study timeline. Before the pandemic, the disease followed a relatively predictable course, characterized predominantly by ascites, moderate hepatic dysfunction, and manageable variceal bleeding episodes. During the pandemic, however, this pattern evolved into acute-on-chronic liver failure, with jaundice, gastrointestinal bleeding, and hepatic encephalopathy becoming more frequent and severe at the time of admission. Similar findings have been reported in international studies, where the pandemic’s healthcare disruptions were associated with delayed hospital presentations and a higher frequency of advanced hepatic decompensation at diagnosis, leading to increased mortality rates among cirrhotic patients [9,29].

During the pandemic, the proportion of Child–Pugh class C cases increased to nearly 50%, while the mean MELD score rose above 21 compared with 18.7 in the pre-pandemic period. These findings are clinically significant, as even a single-point increase in MELD score corresponds to a measurable rise in short-term mortality risk, whereas progression from Child–Pugh class B to C reflects irreversible hepatic synthetic failure. Published evidence supports these observations, indicating that COVID-19–related care delays and infection-associated hepatic injury contributed to accelerated progression and poorer short-term survival in patients with advanced liver disease [3,29].

The Baveno stage, integrating measures of portal hypertension and systemic decompensation, did not differ significantly in distribution but became markedly more predictive of mortality, suggesting a qualitative worsening of portal hypertensive physiology despite similar staging prevalence. Comparable results have been described in European multicenter analyses, where increased portal hypertension severity and systemic inflammation were identified as key predictors of poor outcomes in post-COVID cirrhotic cohorts [9,16,17].

Although the overall prevalence of symptoms such as abdominal pain, ascites, or edema remained relatively stable across the study periods, their prognostic relevance shifted markedly. The pandemic phase was distinguished by a sharp increase in cases presenting with severe jaundice, which emerged as an independent predictor of in-hospital mortality. These findings likely reflect delayed healthcare-seeking behavior: many patients postponed hospital presentations because of fear of SARS-CoV-2 infection or due to reduced hepatology service capacity. As a result, admissions occurred predominantly in late, irreversible stages of decompensation, where urgent interventions such as endoscopy or paracentesis were more often indicated but performed less frequently [16,17].

Hospital stay, ICU transfer, and associated costs provide a sharp functional perspective on how the pandemic reshaped hospital dynamics. The reduction in average length of stay during 2020, paralleled by an increase in ICU admissions, reveals a systemic prioritization of patient throughput and infection control over prolonged stabilization. Despite shorter admissions, hospitalization costs rose by nearly 13%, reflecting the cumulative burden of high-acuity management, personal protective measures, and isolation infrastructure [33,34].

In the post-pandemic period, both length of stay and costs gradually approached pre-pandemic norms; however, the surge in transfusion rates (47.1% vs. 34.4%) points to a rebound phenomenon—patients who had deferred evaluation or treatment during lockdowns often returned in advanced, hemorrhagic, or decompensated states demanding intensive resource use [30]. This pattern exemplifies a broader principle highlighted in recent analyses: even short-term interruptions in hepatology and chronic disease management can precipitate lasting escalations in disease severity and cost [35].

Mortality increased markedly from 21.7% in the pre-pandemic period to 30.2% during the pandemic (*p* = 0.044), representing a 39% relative rise with both statistical and clinical significance. This escalation likely reflects the compounded impact of deferred medical evaluation, restricted endoscopic access, and the predominance of late-stage presentations requiring emergency intervention. Even in the post-pandemic interval, mortality persisted at 26.4%, underscoring that healthcare systems have not yet fully recovered their pre-crisis capacity for timely cirrhosis management. Comparable international analyses reported mortality surges ranging from 25% to 45% among hospitalized cirrhotic patients during COVID-19 peaks [9,29]. Collectively, these findings reinforce that the pandemic’s lethality extended far beyond direct viral infection—its disruption of care pathways and procedural continuity effectively redefined chronic liver disease outcomes worldwide [36,37,38].

### 4.3. Regression Models and Integrated Prognostic Interpretation

The multivariate logistic regression models provide a more nuanced perspective on the pathophysiological and systemic dynamics driving mortality trends. When integrating all study variables, these analyses illustrate how baseline disease severity and acute decompensatory events interacted in distinct ways throughout the different study periods.

When all study periods were evaluated collectively, the Child–Pugh, MELD, and Baveno scores remained robust predictors of in-hospital mortality (*p* < 0.001). Each incremental point in the Child–Pugh score corresponded to nearly a 50% increase in death risk, while each additional MELD point elevated mortality odds by approximately 11%. The Baveno stage demonstrated an odds ratio (OR) of 12.4, reinforcing its comprehensive value in capturing the hemodynamic and systemic consequences of portal hypertension, ascites, variceal bleeding, and hepatic encephalopathy.

Beyond these established prognostic indicators, the model also identified clinical jaundice (OR = 38.1), abdominal pain (OR = 4.3), splenomegaly (OR = 3.6), and the need for paracentesis (OR = 5.4) as significant independent mortality determinants. These associations underline that outcomes in decompensated cirrhosis are driven not only by chronic hepatic insufficiency but also by acute systemic congestion and inflammatory stress [39,40]. The requirement for paracentesis often signals refractory ascites, renal dysfunction, or spontaneous bacterial peritonitis, while splenomegaly and jaundice reflect chronic portal hypertension and impaired bilirubin clearance, bridging the functional and structural dimensions of terminal liver disease [41,42].

Before the COVID-19 pandemic, mortality in decompensated cirrhosis was largely predictable and dictated by baseline hepatic reserve rather than acute events. Only the Child–Pugh (OR = 1.76) and Baveno stage (OR = 21.6) showed statistical significance, reflecting a stable equilibrium between chronic liver dysfunction and effective, timely medical intervention within well-functioning healthcare systems. This period was characterized by early detection, consistent endoscopic access, and manageable complications that rarely translated into fatal outcomes [9].

During the pandemic, however, the prognostic landscape changed profoundly. Upper gastrointestinal bleeding (OR = 355) and jaundice (OR = 294) emerged as dominant mortality predictors, surpassing traditional indices such as the Child–Pugh score, which lost statistical weight. These findings highlight how system-level disruptions—reduced endoscopy availability, blood shortages, and delayed admissions—transformed treatable complications into life-threatening emergencies [16,40]. The prominence of jaundice as a predictor underscores the cumulative impact of systemic inflammation, secondary infections, and delayed therapeutic intervention in advanced hepatic decompensation [3,43].

In the post-pandemic phase, the regression model demonstrated a partial return to pre-crisis dynamics. The Child–Pugh (OR = 2.13), Baveno (OR = 31.9), and MELD (OR = 1.16) scores once again emerged as stable, independent predictors of mortality, reflecting the gradual restoration of clinical pathways and improved continuity of hepatology care. Yet, this recovery was only partial—the persistence of elevated odds ratios suggests that systemic strain and delayed care continued to exert lingering effects on patient outcomes [9,28,29].

Although readmission rates were not evaluated in the present study, the relationship between reduced hospital length of stay and early re-decompensation deserves consideration. In pre-pandemic cohorts of patients with decompensated cirrhosis, 30-day readmission rates were typically reported between 20–30%, with 90-day rates approaching 30–40% [44]. During the pandemic, analyses focusing on patients without active SARS-CoV-2 infection described either modest increases or largely unchanged short-term readmission rates, generally ranging from approximately 25–35%, with differences not consistently reaching statistical significance [18,28]. Importantly, these studies emphasized delayed presentation and greater clinical severity rather than a marked surge in readmission frequency. Early post-pandemic observations suggest gradual stabilization of healthcare utilization patterns. Collectively, available data indicate that shorter hospital stays during the pandemic did not uniformly translate into significantly higher short-term readmission among non-COVID cirrhotic patients, although disease acuity at subsequent encounters may have been greater [9,20].

Taken together, these temporal patterns reveal how the pandemic fundamentally redefined the natural history of cirrhosis, transforming what had once been a chronic, clinically manageable disease into an acutely volatile condition, shaped as much by healthcare disruption as by hepatic pathophysiology itself.

### 4.4. Study Limitations

This study has several inherent limitations that should be acknowledged. Its retrospective, single-center design may limit the generalizability of the findings, as differences in clinical practice, physician experience, and healthcare resource allocation may exist across institutions and even among treating teams within the same hospital. Moreover, although the study period encompassed distinct phases of the COVID-19 pandemic, unmeasured confounders—such as evolving admission policies, treatment availability, and patient health-seeking behavior—could have influenced the results. The economic evaluation relied on hospital billing data, which may not fully capture indirect or societal costs.

Nevertheless, the study’s main strength lies in its temporal design, which enabled the comparative assessment of risk factor dynamics across three well-defined periods—pre-pandemic, pandemic, and post-pandemic—providing novel insight into how systemic healthcare disruptions reshaped the clinical evolution and outcomes of patients with decompensated cirrhosis.

## 5. Conclusions

This study demonstrates that the COVID-19 pandemic fundamentally reshaped the clinical trajectory of patients with decompensated liver cirrhosis. Distinct temporal shifts in disease severity, complication rates, and mortality highlight how systemic healthcare strain transformed a predictable chronic condition into an acutely unstable syndrome. During the pandemic, restricted access to diagnostic procedures, delayed presentation, and limited critical care capacity significantly worsened clinical outcomes and increased in-hospital mortality.

Although classical prognostic indices—Child–Pugh, MELD, and Baveno scores—remained strong predictors of mortality across all phases, their relative impact varied according to healthcare accessibility. The persistence of elevated MELD scores and mortality rates even after the pandemic indicates only partial recovery of hepatology services. Together, these findings illustrate a continuum of systemic vulnerability—from stability before the pandemic, through collapse during it, to incomplete restoration afterward—and underscore the urgent need to strengthen healthcare resilience and continuity for patients with chronic liver disease in the face of future crises.

## Figures and Tables

**Table 1:** Demographic aspects.

Variables	Pre-Pandemic (*n* = 138)	Pandemic (*n* = 96)	Post-Pandemic (*n* = 121)	*p*
Age (years, M ± SD)	59.93 ± 10.27	59.66 ± 10.39	58.72 ± 10.65	0.632 *
Gender Male	109 (79%)	79 (82.3%)	89 (73.6%)	0.286 **
Environment				
Urban	74 (53.6%)	48 (50%)	62 (51.2%)	0.851 **
Rural	64 (46.4%)	48 (50%)	59 (48.8%)	

M = mean; SD = Standard deviation. * *p*-values were calculated using ANOVA; ** *p*-values were calculated using the Chi-square test.

**Table 2:** Clinical reasons of presentation.

Variables	Pre-Pandemic (*n* = 138)	Pandemic (*n* = 96)	Post-Pandemic (*n* = 121)	*p*
UGIB	32 (23.2%)	18 (18.8%)	36 (29.8%)	0.160
LGIB	4 (2.9%)	2 (2.1%)	4 (3.3%)	0.862
LLE	15 (10.9%)	9 (9.4%)	9 (7.4%)	0.637
Jaundice	16 (11.6%)	13 (13.5%)	15 (12.4%)	0.906

This table summarizes the variation in the presence of the most frequently reported symptoms at admission. As patients could present with multiple concomitant symptoms or others less frequent, the reported percentages do not total 100%. *p*-values were calculated using the Chi-square test.

**Table 3:** Cirrhosis etiology.

Cirrhosis Etiology	Pre-Pandemic (*n* = 138)	Pandemic (*n* = 96)	Post-Pandemic (*n* = 121)	*p*
Alcoholic	112 (81.2%)	73 (76%)	92 (76%)	0.873
Mixed	8 (5.8%)	9 (9.4%)	13 (10.7%)	
Viral	14 (10.1%)	11 (11.5%)	13 (10.7%)	
others	4 (2.9%)	3 (3.1%)	3 (2.5%)	

*p*-values were calculated using the Chi-square test.

**Table 4:** Child Pugh variation across the 3 periods.

Child Pugh Class	Pre-Pandemic (*n* = 138)	Pandemic (*n* = 96)	Post-Pandemic (*n* = 121)	*p*
A	17 (12.3%)	7 (7.3%)	15 (12.3%)	0.011
B	59 (42.8%)	42 (43.8%)	57 (47.1%)	
C	62 (44.9%)	47 (49%)	49 (40.49%)	

*p*-values were calculated using the Chi-square test.

**Table 5:** Baveno variation across the 3 periods.

BAVENO	Pre-Pandemic (*n* = 138)	Pandemic (*n* = 96)	Post-Pandemic (*n* = 121)	*p*
4	58 (42%)	37 (38.5%)	58 (47.9%)	0.657
5	59 (42.8%)	41 (42.7%)	44 (36.4%)	
6	21 (15.2%)	18 (18.8%)	19 (15.7%)	

*p*-values were calculated using the Chi-square test.

## Data Availability

The original contributions presented in the study are included in the article. Further inquiries can be directed to the corresponding author.

## Supplementary Materials

The following supporting information can be downloaded at: https://www.mdpi.com/article/10.3390/medicina62020404/s1, Table S1: Independent predictive factors for in-hospital mortality across all three study periods; Table S2: Independent predictive factors for in-hospital mortality for pre-pandemic period; Table S3: Independent predictive factors for in-hospital mortality for pandemic period; Table S4: Independent predictive factors for in-hospital mortality for post-pandemic period.
