# Supplementary material for: Assessing the Long-Term Impact of the COVID-19 Pandemic on Hospital Outcomes in Patients with Decompensated Liver Cirrhosis
Source: Medicina (Kaunas). 2026 Feb 19;62(2):404. doi: 10.3390/medicina62020404 (PMC12942391; doi:10.3390/medicina62020404)
Supplement: Supplementary file 1 [file medicina-62-00404-s001.zip › medicina-4139890-supplementary.pdf]

Table S1. Independent predictive factors for in-hospital mortality across all three study periods.

Variables in the Equation									
		B	S.E.	Wald	df	Sig.	Exp(B)	95% C.I.for EXP(B)	
								Lower	Upper
Step 1[a]	Child-Pugh	.409	.128	10.237	1	.001	1.505	1.172	1.933
	BAVENO	2.519	.405	38.767	1	.000	12.421	5.620	27.452
	MELD	.107	.030	13.002	1	.000	1.113	1.050	1.180
	Esophageal varices	-.340	.801	.181	1	.671	.711	.148	3.421
	Splenomegaly	1.295	.515	6.314	1	.012	3.652	1.330	10.029
	Ascites	-.190	.549	.120	1	.729	.827	.282	2.423
	Abdominal pain	1.462	.478	9.344	1	.002	4.316	1.690	11.023
	UGIB	.977	.554	3.118	1	.077	2.657	.898	7.863
	LGIB	1.428	1.249	1.308	1	.253	4.171	.361	48.209
	Jaundice	3.642	.904	16.225	1	.000	38.161	6.487	224.492
	Paracentesis	1.688	.653	6.675	1	.010	5.410	1.503	19.474
	Constant	-25.899	3.512	54.375	1	.000	.000		
a. Variable(s) entered on step 1: Child-Pugh, BAVENO, MELD, Esophageal varices, Splenomegaly, Ascites, Abdominal pain, UGIB= upper gastrointestinal bleeding; LGIB= lower gastrointestinal bleeding; Jaundice, Paracentesis									

Table S2. Independent predictive factors for in-hospital mortality for pre-pandemic period.

Variables in the Equation									
		B	S.E.	Wald	df	Sig.	Exp(B)	95% C.I.for EXP(B)	
								Lower	Upper
Step 1[a]	Child-Pugh	.565	.262	4.651	1	.031	1.759	1.053	2.939
	BAVENO	3.074	.980	9.846	1	.002	21.624	3.170	147.489
	MELD	.050	.061	.678	1	.410	1.051	.933	1.184
	Esophageal varices	.477	1.549	.095	1	.758	1.612	.077	33.552

	Splenomegaly	1.494	1.085	1.896	1	.169	4.457	.531	37.396
	Ascites	-1.042	1.129	.852	1	.356	.353	.039	3.226
	Abdominal pain	1.670	.934	3.196	1	.074	5.311	.851	33.128
	UGIB	-.838	.999	.703	1	.402	.433	.061	3.067
	LGIB	-.487	1.771	.076	1	.783	.615	.019	19.772
	Jaundice	33.226	10479.557	.000	1	.997	26911772873 8639.720	.000	.
	Paracentesis	1.205	1.321	.832	1	.362	3.338	.251	44.463
	Constant	-44.370	7194.326	.000	1	.995	.000		

a. Variable(s) entered on step 1: Child-Pugh, BAVENO, MELD, Esophageal varices, Splenomegaly, Ascites, Abdominal pain, UGIB= upper gastrointestinal bleeding; LGIB= lower gastrointestinal bleeding; Jaundice, Paracentesis

Table S3. Independent predictive factors for in-hospital mortality for pandemic period.

Variables in the Equation									
		B	S.E.	Wald	df	Sig.	Exp(B)	95% C.I.for EXP(B)	
								Lower	Upper
Step 1[a]	Child-Pugh	.475	.340	1.949	1	.163	1.608	.825	3.132
	BAVENO	3.411	1.025	11.082	1	.001	30.300	4.066	225.766
	MELD	.159	.079	4.122	1	.042	1.173	1.006	1.368
	Esophageal varices	-4.065	2.372	2.937	1	.087	.017	.000	1.793
	Splenomegaly	.975	1.132	.741	1	.389	2.650	.288	24.385
	Ascites	1.888	1.208	2.443	1	.118	6.608	.619	70.536
	Abdominal pain	2.667	1.465	3.312	1	.069	14.394	.814	254.410
	UGIB	5.873	2.008	8.558	1	.003	355.287	6.946	18171.812
	LGIB	16.106	26172.71 6	.000	1	1.000	9878079.856	.000	.
	Jaundice	5.684	2.086	7.427	1	.006	293.981	4.933	17520.171
	Paracentesis	2.218	1.625	1.863	1	.172	9.185	.380	221.842

	Constant	-67.786	32425.073	.000	1	.998	.000		
a. Variable(s) entered on step 1: Child-Pugh, BAVENO, MELD, Esophageal varices, Splenomegaly, Ascites, Abdominal pain, UGIB= upper gastrointestinal bleeding; LGIB= lower gastrointestinal bleeding; Jaundice, Paracentesis									

Table S4. Independent predictive factors for in-hospital mortality for post-pandemic period.

**Variables in the Equation**									
		B	S.E.	Wald	df	Sig.	Exp(B)	95% C.I.for EXP(B)	
								Lower	Upper
Step 1[a]	Child-Pugh	.757	.287	6.940	1	.008	2.131	1.214	3.742
	BAVENO	3.463	.946	13.411	1	.000	31.928	5.002	203.796
	MELD	.147	.062	5.580	1	.018	1.158	1.025	1.309
	Esophageal varices	.924	1.595	.335	1	.562	2.519	.111	57.365
	Splenomegaly	2.026	1.131	3.206	1	.073	7.580	.826	69.599
	Ascites	.171	1.192	.020	1	.886	1.186	.115	12.269
	Abdominal pain	.385	.964	.159	1	.690	1.469	.222	9.716
	UGIB	.312	1.224	.065	1	.799	1.366	.124	15.050
	LGIB	5.875	2.562	5.256	1	.022	355.896	2.345	54010.904
	Jaundice	2.984	2.729	1.196	1	.274	19.764	.094	4153.376
	Paracentesis	3.067	1.662	3.406	1	.065	21.486	.827	558.474
	Constant	-38.467	9.485	16.447	1	.000	.000		
a. Variable(s) entered on step 1: Child-Pugh, BAVENO, MELD, Esophageal varices, Splenomegaly, Ascites, Abdominal pain, UGIB= upper gastrointestinal bleeding; LGIB= lower gastrointestinal bleeding; Jaundice, Paracentesis									
